# MicroRNA Drop in the Bloodstream and MicroRNA Boost in the Tumour Caused by Treatment with Ribonuclease A Leads to an Attenuation of Tumour Malignancy

**DOI:** 10.1371/journal.pone.0083482

**Published:** 2013-12-30

**Authors:** Nadezhda Mironova, Olga Patutina, Evgenyi Brenner, Alexander Kurilshikov, Valentin Vlassov, Marina Zenkova

**Affiliations:** Laboratory of Nucleic Acids Biochemistry, Institute of Chemical Biology and Fundamental Medicine, Siberian Branch of Russian Academy of Sciences, Novosibirsk, Russian Federation; Sapporo Medical University, Japan

## Abstract

Novel data showing an important role of microRNAs in mediating tumour progression opened a new field of possible molecular targets for cytotoxic ribonucleases. Recently, antitumour and antimetastatic activities of pancreatic ribonuclease A were demonstrated and here genome-wide profiles of microRNAs in the tumour and blood of mice bearing Lewis lung carcinoma after treatment with RNase A were analysed by high-throughput Sequencing by Oligonucleotide Ligation and Detection (SOLiD™) sequencing technology. Sequencing data showed that RNase A therapy resulted in the boost of 116 microRNAs in tumour tissue and a significant drop of 137 microRNAs in the bloodstream that were confirmed by qPCR. The microRNA boost in the tumour was accompanied by the overexpression of microRNA processing genes: RNASEN (Drosha), xpo5, dicer1, and eif2c2 (Ago2). Ribonuclease activity of RNase A was shown to be crucial for the activation of both microRNA synthesis and expression of the microRNA processing genes. In the tumour tissue, RNase A caused the upregulation of both oncomirs and tumour-suppressor microRNAs, including microRNAs of the let-7 family, known to negatively regulate tumour progression. Our results suggest that the alteration of microRNA signature caused by RNase A treatment leads to the attenuation of tumour malignancy.

## Introduction

During the last decade, at the forefront of molecular and cellular biology is the identification and study of non-coding RNAs, particularly microRNAs (miRNAs). miRNAs have emerged as a novel class of potent regulatory molecules that enforce post-transcriptional silencing of gene expression through the RNA interference pathway [1]–[4], and are responsible for the modulation of fundamental physiological processes inside the cell and at the level of the whole organism [5]–[7]. Ample studies suggest that the dysregulation of individual miRNAs or miRNA families is associated with the initiation of pathogenesis and progression of a wide spectrum of diseases, including oncology [8], [9]. Novel miRNA-targeting oligonucleotide-based tools have been developed and progress was achieved with a cure of specific diseases by turning off certain miRNAs [10]–[12].

Tumour development is accompanied by significant changes in the expression of multiple sets of miRNAs, which display oncogenic and tumour-suppressing properties [8], [9]. Due to the multiplicity and complexity of miRNA expression defects in cancer, miRNA networks can be considered as prospective therapeutic targets.

The important role of miRNA in the regulation of tumourigenesis suggests that enzymes capable of cleaving RNA are promising anticancer drugs. Antitumour potential is known since long ago for exogenous ribonucleases (RNases) derived from fungi (streptomycete ribonuclease [13]), bacteria (binase [14]–[16]), reptiles (onconase [17]–[19], cSBL and jSBL RNases [20]), and mammals [21]–[24]. A number of investigations were devoted to the direct cytotoxic effect of natural ribonucleases on tumour cells associated with intracellular coding and non-coding RNA cleavage [25]–[30]. At the same time, no studies have been conducted to evaluate the potential alterations in tumour-specific and/or circulating miRNA profiles and investigate the association of miRNA expression pattern and therapeutic efficacy of treatment with ribonucleases *in vivo*.

Recently, using two murine tumour models, we have shown that the pancreatic ribonuclease RNase A is capable of retarding primary tumour growth and efficiently inhibiting the development of metastases [22]–[24]. Taking into account the high ribonucleolytic activity of RNase A [31] and given the fact that RNase A cannot have a direct cytotoxic effect on tumour cells by the cleavage of intracellular RNAs, due to its binding with cytosolic ribonuclease inhibitor [32], [33], we hypothesised that the enzyme affects the regulatory pathways of tumourigenesis through the cleavage of circulating oncogenic miRNAs and alteration of miRNA profile.

Here, in order to find molecular targets of RNase A, we performed an analysis of genome-wide profiles of miRNAs in the tumour and serum of mice bearing Lewis lung carcinoma (LLC), after treatment with RNase A by high-throughput Sequencing by Oligonucleotide Ligation and Detection (SOLiD™) sequencing technology. We found that antitumour and antimetastatic activities of RNase A were associated with the change of miRNA profiles in the blood serum and tumour tissue and with the alteration of the miRNA signature of the tumour from malignant to more normal.

## Materials and Methods

### Tumour transplantation and design of animal experiment

All animal procedures were carried out in strict accordance with the recommendations for proper use and care of laboratory animals (ECC Directive 86/609/EEC). The protocol was approved by the Committee on the Ethics of Animal Experiments of the Administration of Siberian Branch of Russian Academy of Sciences.

Twelve- to 14-week-old female C57Bl/6J mice were used. LLC in solid form was induced by intramuscular (i.m.) injection of tumour cells (10^6^) suspended in 0.1 ml of saline buffer into the right thighs of mice. On day 4 after tumour transplantation, mice were treated with saline buffer (n = 30) or RNase A (Sigma, USA) at a dose of 0.7 µg/kg (n = 20). RNase A was administered in a volume of 0.1 ml i.m. daily except for weekends. The total number of injections was 10. Tumour size was monitored every other day using caliper measurements in three perpendicular dimensions. Tumour volumes were calculated as V = (π/6×length×width×height). On day 15 after tumour transplantation, blood and tumour samples were collected 60 min after the last injection of RNase A.

### Inactivation of RNase A by diethyl pyrocarbonate

A reaction mix containing 5×10^−4^ M RNase A in a volume of 300 µl and 6 µl diethyl pyrocarbonate (DEPC) (Sigma) was incubated at room temperature for 20 h under constant shaking. At 6 and 12 h after the beginning of incubation, 3 µl DEPC was repeatedly added. After the completion of the reaction, residual DEPC was eliminated by incubating the reaction mix at 37°C for 90 min.

### Preparation of primary cell culture of LLC and treatment with RNase A

The isolation of primary cell culture from LLC solid tumours was carried out by homogenisation of tumour tissue and separation of living cells from the total cell suspension using Lymphocyte Separation Medium (LSM) (ICN, USA). LLC cells were seeded in triplicate on 6-well plates at a density of 1.5×10^6^ cells/well in Iscove's Modified Dulbecco's Medium (IMDM) (Sigma) supplemented with 10% foetal bovine serum (FBS) (Sigma), 1% antibiotic antimycotic solution (10.000 µg/mL streptomycin, 10.000 IU/mL penicillin, and 25 µg/mL amphotericin) (ICN). Saline buffer, RNase A, or DEPC-treated RNase A was added to the cell suspension to a final concentration of 10^−8^ M. Cells were incubated at 37°C and 5% CO_2_ for 48 h prior to RNA isolation.

### Sample processing and RNA extraction

Tumour pieces from mice treated with saline buffer or RNase A were pooled according to groups and homogenised, and total RNA was extracted immediately using TRIzol Reagent (Invitrogen, USA). The fractionation of isolated RNA to short (<200 nt) and long (>200 nt) RNA pools was performed using the mirVana miRNA Isolation Kit (Ambion, USA) according to the manufacturer's protocols. Total RNA was extracted from primary cell culture of intact LLC and LLC treated with RNase A using TRIzol Reagent according to the manufacturer's protocol.

Blood serum was prepared from the whole blood of animals treated with saline buffer or RNase A by clot formation at 37°C for 30 min and at 4°C overnight, followed by clot discard and centrifugation (4000 rpm, 4°C, 20 min) to remove cell debris. Serum samples of 30 control animals, which received saline buffer, and 20 experimental animals, which received RNase A, were pooled according to groups. The total RNA including small RNA fraction was extracted from 4 ml of serum of control animals (half of the total serum volume) and 5.5 ml of serum of experimental animals (total serum volume) using TRIzol Reagent according to a modified method (serum/TRIzol/chloroform, 1/3/0.1, v/v/v, centrifugation at 12,500 rpm for 15 min, 4°C). Total RNA integrity and quantity were checked using Bioanalyzer (Agilent Technologies, USA). The enrichment of serum miRNA fraction was performed using the mirVana miRNA Isolation Kit.

The following fractions of RNA were obtained: tumour RNA fractions – long RNA fraction isolated from tumour tissue of control mice treated with saline buffer (RNA_LTc_), long RNA fraction isolated from tumour tissue of mice treated with RNase A (RNA_LTR_), short RNA fraction isolated from tumour tissue of control mice treated with saline buffer (RNA_STc_), short RNA fraction isolated from tumour tissue of mice treated with RNase A (RNA_STR_); serum RNA fractions – short RNA fraction isolated from blood serum of control mice treated with saline buffer (RNA_SSc_), short RNA fraction isolated from blood serum of mice treated with RNase A (RNA_SSR_); and total RNA fraction – total RNA isolated from control cell culture of LLC (RNA_Tc_), total RNA isolated from cell culture of LLC treated with RNase A (RNA_TR_).

### Small RNA library preparation and sequencing

Libraries of small RNAs were prepared by the SOLiD™ small RNA expression kit, SREK (Applied Biosystems, USA) in accordance with the manufacturer's recommendations. In brief, short RNA fractions RNA_STc_, RNA_STR_, RNA_SSc_, and RNA_SSR_ (100 ng of each) were mixed with the adapter mix A and incubated for ligation for 16 h at 4°C, followed by reverse transcription (RT) and RNase H treatment. Large-scale PCRs were performed using 17 cycles both for tumour and serum samples. The obtained adaptor-ligated PCR products were then size-selected (105–150 bp) using 6% PAGE gel, and eluted from the gel by repeated heating at 70°C for 5 min and freezing at −20°C for 10 min, followed by incubation in the presence of 3 M ammonium acetate at 45°C overnight. Desalting of the eluted PCR products was performed using the QIAquick Gel Extraction Kit (Qiagen, Germany). Then, the PCR products were quantified using the Quant-iT™ dsDNA HS Assay Kit (Invitrogen). The absence of PCR by-products of shorter than 105 bp (e.g., adaptor dimers) was verified using the High Sensitivity DNA Assay for Agilent 2100 Bioanalyzer (Agilent Technologies). Templated bead preparation, emulsion PCR, and deposition were performed, following the standard SOLiD™ V3.5 (Applied Biosystems) protocols.

All four samples were run on a single slide, each on its quadrant, with 35 nucleotides read length on the SOLiD™ 3.5 system resulting, accordingly, in 7.17×10^7^, 5.14×10^7^, 4.26×10^7^, and 5.65×10^7^ total number of reads.

Sequencing data have been submitted to the NIH Short Read Archive (accession number SRP031771).

### qPCR

The study of alteration in miRNA expression was performed using stem-loop PCR technology [34], [35]. miRNA cDNA synthesis was carried out using SuperScript III reverse transcriptase (Invitrogen). The reaction of reverse transcription was performed in a total volume of 20 µl containing 3 µg of RNA_STc_, RNA_STR_, RNA_Tc_, or RNA_TR_ or 0.25 µg of RNA_SSc_ or RNA_SSR_, 1× First-Strand Buffer, 0.5 mM dNTPs, 10 mM DTT, 200 units of SuperScript III reverse transcriptase, and 0.05 µM of miRNA-specific stem-loop primers (Table S1). RT primers were preheated at 70°C for 5 min and incubated on ice for 2 min before being added to the reaction mix. RT was accomplished with initial incubation at 16°C for 30 min followed by gradual annealing and extension for 60 cycles at 30°C for 30 s, 42°C for 30 s, and 50°C for 30 s, and a final reverse transcriptase inactivation at 85°C for 5 min. PCR amplification was carried out in a total volume of 20 µl using 1× Advantage 2 Polymerase Mix (Clontech, USA), 1× Advantage 2 PCR Buffer, 0.6 mM dNTPs, 1× EvaGreen (Biotium, Hayward, USA), and 0.2 µM of PCR sense and antisense primers (Table S2). The reaction was performed with initial preheating at 95°C for 2 min followed by 40 cycles of denaturing at 94°C for 15 s, annealing at 58°C for miR-21, miR-29b-1, let7-g, miR-10b, miR-451a, miR-17, and miR-18a, or 62°C for miR-145a and miR-31 for 30 s, and elongation at 70°C for 30 s. The expression of tumour-derived miRNAs was rated relatively to *U6* and *rpl30* and the concentration of serum-derived miRNAs was normalised to serum volume.

Expression levels of mRNAs encoding miRNA processing proteins Drosha, Xpo5, Dicer1, and Eif2c2 (Argonaute 2 [Ago2]) were evaluated using qPCR. cDNA synthesis was performed in a total volume of 40 µl containing 5 µg of RNA_LTc_, RNA_LTr_, RNA_Tc_, or RNA_Tr_, 1×RT Buffer (50 mM Tris-HCl, pH 8.3, 75 mM KCl, 3 mM MgCl_2_), 10 mM DTT, 0.5 mM dNTPs, 100 pmol random hexa-primers, and 20 units of M-MLV reverse transcriptase. The reaction was carried out at 37°C for 60 min. The obtained RT reaction was used to prepare serial dilutions with factors of 10^−2^, 10^−3^, and 10^−4^. In order to evaluate dynamic range and PCR efficacy, 5 µl of each dilution was used per qPCR reaction. PCR amplification was carried out in a total volume of 20 µl using 0.5 units of AmpliTaq Gold (Applied Biosystems), 1×PCR Gold Buffer, 2.5 mM MgCl_2_, 0.35 mM dNTPs, 1× EvaGreen (Biotium), and 0.25 µM PCR sense and antisense primers (Table S2). The reaction was performed with initial preheating at 94°C for 6 min followed by 40 cycles of denaturing at 94°C for 12 s, annealing at 60°C for 15 s, and elongation at 72°C for 30 s. *hprt1* and *rpl30* were chosen as reference genes from the following set: *gapdh*, *ubc*, *18S rRNA*, *rpl30*, *hprt1*, and *ywhaz*, as having a minimal M-Value. Relative gene expression was calculated using the standard BioRad IQ5 software (BioRad, USA).

### Statistics

To determine the differential expression in samples, the reads were mapped to the *Mus musculus* NCBI37 genome using a complex mapping scheme with iterative reduction of seed length from 25 to 16 and a preliminary filtering step; 2.63×10^7^, 1.87×10^7^, 1.39×10^7^, and 1.78×10^7^ reads respective to the libraries were mapped to the genome uniquely. Mapping was performed using the Bioscope v.1.3 software package (Life Technologies, USA). Feature extraction, reads per kilobase per million reads in library (RPKM, where RPKM = number of reads of specific miRNA/(size of miRNA(kb) × total number of reads in library(mln))) calculation, and statistical analysis was performed using the Cufflinks v.2.0.1 software (Center for Bioinformatics and Computational Biology, USA).

qPCR data were statistically processed using Student's t-test (two-tailed, unpaired); a *p* value of ≤0.05 was considered to indicate a significant difference.

## Results

**Figure 1:**
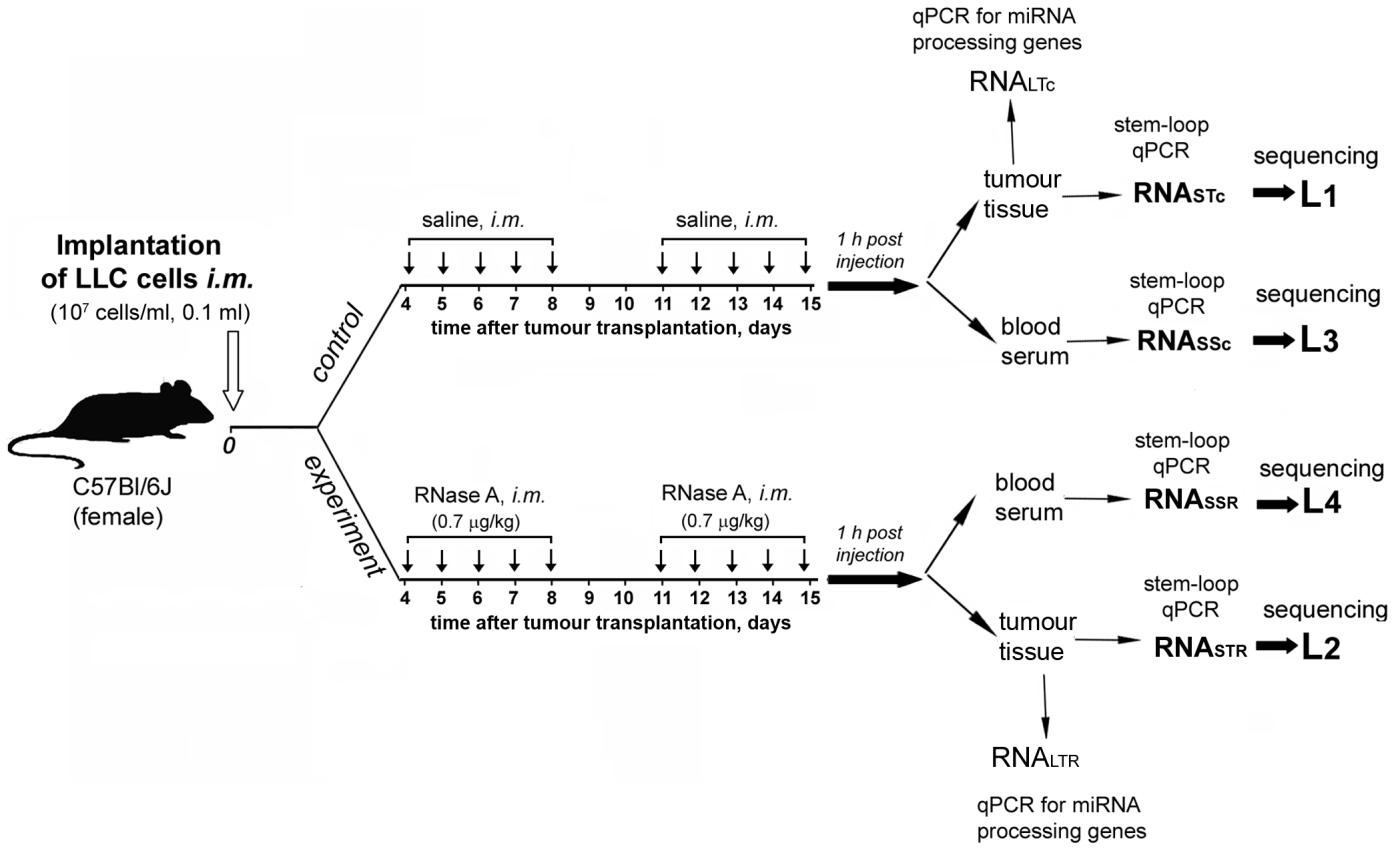
Scheme of the experiment for cDNA library preparation. Mice with intramuscularly (i.m.) implanted LLC were treated with saline or RNase A at a dose of 0.7 µg/kg for 10 days starting on the 4^th^ day after tumour transplantation. At 1 h after the last injection, tumour tissue and blood serum samples were collected and pooled according to groups, and long and short RNA fractions were isolated. Long RNA fractions RNA_LTc_ and RNA_LTR_ were used for qPCR for the evaluation of the expression levels of miRNA processing genes. Short tumour-derived (RNA_STc_ and RNA_STR_) and serum-derived (RNA_SSc_ and RNA_SSR_) RNA fractions were used for the preparation of cDNA libraries and subsequent sequencing on the SOLiD™ ABA 3.5 platform and for stem-loop qPCR.

To study the possible mechanism of RNase A-mediated antitumour effect we evaluated the alteration of miRNA profiles in the tumour tissue and blood serum of mice with LLC after treatment with the enzyme. The experimental scheme is shown in Figure 1. Two groups of mice with i.m. implanted LLC were treated with saline buffer or RNase A according to the previously developed scheme (see Materials and Methods) [22]. Tumour and serum samples were collected and pooled according to the type of samples within one group, and then long and short RNA fractions were isolated. The obtained pooled tumour-derived (RNA_STc_ and RNA_STR_) and serum-derived miRNA samples (RNA_SSc_ and RNA_SSR_) were used for the preparation of cDNA libraries and subsequent sequencing on the SOLiD™ ABA 3.5 platform.

### Sequencing data of tumour and serum miRNAs after RNase A therapy

The fragment libraries were generated from pooled tumour- (L1 and L2) and serum-derived RNAs (L3 and L4) of corresponding groups of mice (Figure 1). Table 1 shows the estimation of miRNA in the studied libraries. In total, between 2.6 to 9.3 million reads for every library were classified as miRNA sequences. In tumour and serum libraries, 2089 and 972 differentially expressed genes (including 123 and 139 miRNA genes) were found, respectively.

**Table 1:** Estimation of microRNA (miRNA) enrichment by mapping the sequencing data to the reference.

Library	miRNA*	mRNA*	rRNA*	Filter*	Mapped*	Total, number of reads
L1	965 017 (1.4%)	31 407 252 (43.8%)	9 348 471 (13%)	6 511 041 (9.1%)	48 231 781 (67.3%)	71 675 921
L2	1 399 612 (2.7%)	21 972 560 (42.8%)	5 783 418 (11.3%)	4 986 000 (9.7%)	34 141 590 (66.5%)	51 371 107
L3	835 664 (2.0%)	20 476 883 (48%)	2 623 843 (6.2%)	8 200 681 (19.3%)	32 137 071 (75.5%)	42 561 316
L4	560 568 (1.0%)	27 024 143 (47.8%)	3 195 154 (5.7%)	8 876 496 (15.7%)	39 656 361 (70.2%)	56 516 886

The list of reference sequences includes hairpin miRNA transcripts, matrix RNAs (mRNAs), ribosomal RNAs (rRNAs), “filtering” RNAs (transfer RNAs, repetitive genome elements, SOLiD™ adapter sequences). Mapping was performed by Bioscope v.1.3 with a seed length of 19, allowing two mismatches. cDNA libraries were constructed on the basis of short RNA fractions: L1 – RNA fraction from the tumour tissue of mice with LLC treated with saline buffer (RNA_STc_); L2 – RNA fraction from the tumour tissue of mice with LLC treated with RNase A (RNA_STR_); L3 – RNA fraction from the blood serum of mice with LLC treated with saline buffer (RNA_SSc_); L4 – RNA fraction from the blood serum of mice with LLC treated with RNase A (RNA_SSR_). *- number of reads (percentage of total number mapped on the group of RNAs).

**Figure 2:**
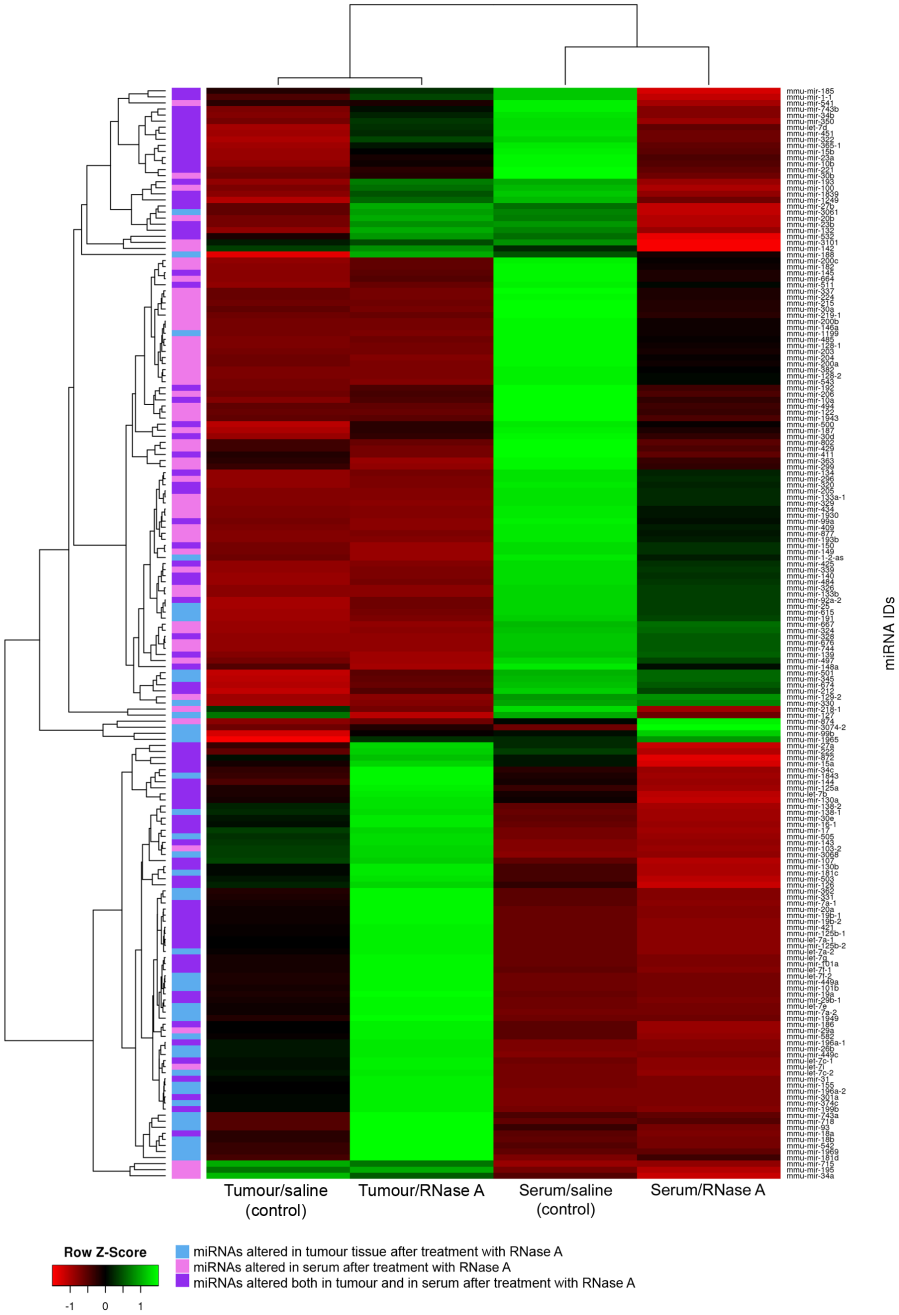
Heat map representing the miRNA profiles in the blood serum and tumour tissue in control and RNase A-treated mice. Dendrograms were derived by pairwise average linkage clustering of miRNA genes or samples using Euclidean distances between row-scaled or column-scaled RPKM values (Z-scores), respectively.

Tumour-derived and serum miRNAs, which showed a reliable change in expression level after treatment with RNase A, are displayed on the Heat map (Figure 2). Sequencing data revealed that treatment with RNase A resulted in an apparent alteration in the levels of 123 out of 615 tumour-derived miRNAs and 139 out of 617 circulating miRNAs (Figure 2, Tables S3 and S4). As we expected, treatment with RNase A resulted in a decrease in the levels of the majority of serum-derived miRNAs: a significant drop of 137 miRNAs and the increase of only 2 miRNAs were observed. To our surprise, RNase A therapy caused noticeable upregulation of miRNAs in tumour tissue: an increase of 116 tumour-derived miRNAs and a decrease of only 7 miRNAs were observed. It is noteworthy that 4 of 7 downregulated tumour-derived miRNAs and 2 increased serum miRNAs had an extremely low number of reads in the sequencing data (Tables S3 and S4). Thus, we found that RNase A treatment led to a global upregulation of miRNAs in the tumour and a significant decrease in the levels of most miRNAs in the blood serum of LLC-bearing mice.

It should be noted that 81 of the affected miRNAs were common to both the tumour and serum (see Figure 2, Tables S3 and S4, highlighted in light purple); 42 miRNAs were tumour-specific (highlighted in light blue) and 58 miRNAs were serum-specific (highlighted in pink). After treatment with RNase A, we observed an alteration in the levels of both common miRNAs and miRNAs specific for the tumour or serum.

The analysis of miRNA profiles showed that the altered pool of miRNAs contained a considerable number of ascertained tumour-associated miRNAs, both oncogenic and tumour-suppressing, such as miRNAs from the let-7 family, mir-107, mir-155, mir-15, mir-16, mir-21, mir-10b, mir-145, mir-451a, mir-29b1, mir-17, mir-18a, and others.

### qPCR validation of miRNA sequencing data

Validation of the alterations in miRNA profiles in the tumour tissue and blood serum after treatment with RNase A was performed using stem-loop PCR technology [34], [35]. The algorithm of miRNA selection for validation consisted of a few steps (Table S5). miRNAs of the L1 library (tumour-derived miRNAs from control animals) and L3 library (serum miRNAs from control animals) were sorted by abundance score (RPKM values) in descending order and the data of the L2 library (tumour-derived miRNAs from animals treated with RNase A) and L4 library (serum miRNAs from animals treated with RNase A) were superposed, respectively. The L2/L1 and L3/L4 fold changes were calculated (the primary data are in Tables S3, S4, and S5). The top 100 miRNAs of the L1 and L2 libraries were selected and sorted by L2/L1 fold change in descending order, and compared with the data of L3/L4. At this step, serum-specific miRNAs that were not discovered in tumour tissue were rejected. Thus, further miRNAs presented in all libraries were analysed.

The final step of the selection consisted of choosing the most abundant and the most affected miRNA species by calculating the total score (TS)/total fold (TF) ratio, where TS = score in L1+ score in L3, and TF = L2/L1 fold change +L3/L4 fold change. Further miRNAs were sorted by TS/TF in ascending order. miRNAs with high TF and TS/TF between 0 to 20 were considered as potential targets for validation. Nine of these miRNAs that were known to be oncomirs or oncosuppressors according to data in the literature were selected for validation using qPCR: *mmu*-mir-29b, *mmu*-mir-21, *mmu*-10b, *mmu*-mir-451a, *mmu*-mir-17, *mmu*-mir-18a, *mmu*-mir-145, *mmu*-mir-31, and *mmu*-let-7g (further in the text without species identity) (Table S5).

**Figure 3:**
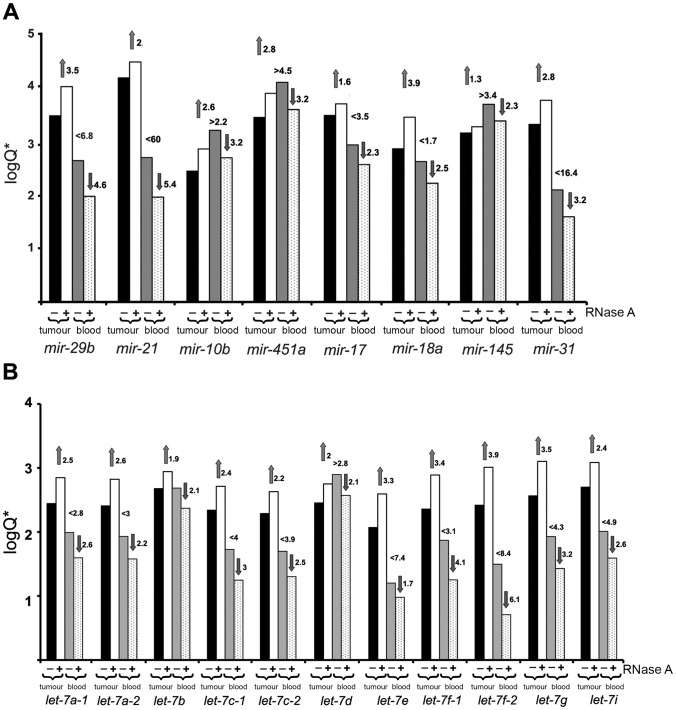
Logarithmic bar-graph depiction of the results of sequencing data. The typical pattern of changes in the levels of some oncogenic and tumour-suppressor miRNAs (**A**), and members of the let-7 miRNA superfamily (**B**) in LLC tumour tissue and serum of tumour-bearing mice after RNase A therapy. Q corresponds to reads per kb per million (RPKM) = number of reads of specific miRNA/(size of miRNA(kb) ×total number of reads in the library(mln)). “−” – mice with LLC treated with saline buffer; “+” – mice with LLC treated with RNase A. Black and white bars reflect miRNA expression in tumour tissue, grey and spotted bars reflect miRNA level in the bloodstream.

To illustrate the sequencing data, the diagram in logarithmic coordinates demonstrating the typical pattern of changes in the levels of selected tumour- and serum-derived miRNAs after treatment with RNase A is shown (Figure 3A). Here, we also found it necessary to show the data on miRNAs of the let-7 family (Figure 3B).

Among the top 50 tumour-derived miRNAs, a 6- to 10-fold increase in the expression of a particular miRNA was observed. In the bloodstream, the decrease of the same miRNAs was 2- to 6-fold. It is worth noting that all miRNAs from the let-7 family known to be tumour suppressors were upregulated in the tumour (Figure 3B).

**Figure 4:**
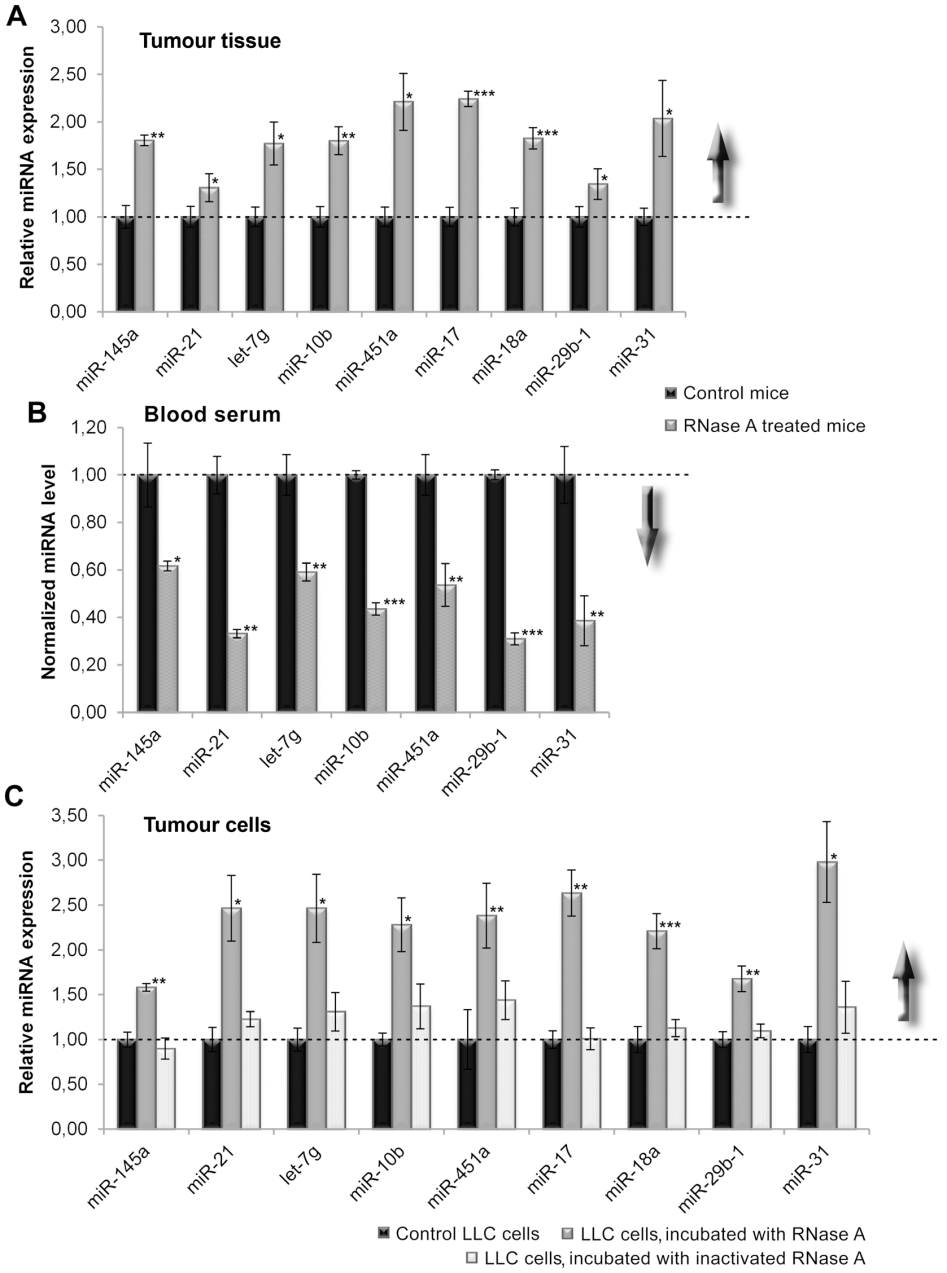
Stem-loop RT-qPCR analysis of miRNA expression levels in tumour cells and blood serum of tumour-bearing mice after treatment with RNase A. A. miRNA expression in tumour tissue from mice with LLC that received RNase A therapy. The expression of miRNAs was normalised to *U6*. B. miRNA levels in the blood serum of mice with LLC that received RNase A therapy. The concentration of serum miRNAs was normalised to serum volume. C. miRNA expression in LLC cells incubated *in vitro* with intact and DEPC-inactivated RNase A for 48 h. The expression of miRNAs was normalised to *U6* and *rpl30*. *, **, and *** denote a statistically significant difference relative to the control with *p*<0.05, *p*<0.01, and *p*<0.001, respectively.

qPCR analysis confirmed the discovered tendency for an increase in the levels of miRNAs in the tumour and decrease in the levels of miRNAs in the serum after treatment with RNase A. An approximate two-fold increase in miRNA level in the tumour tissue (Figure 4A) and an approximate two-fold reduction in miRNA level in the blood serum were observed (Figure 4B).

### The effect of RNase A on miRNA expression in LLC primary cell culture

To confirm the stimulating effect of RNase A on miRNA expression in tumour cells, an *in vitro* experiment was performed using a primary cell culture of LLC exposed to RNase A or to inactivated (DEPC-treated) RNase A at a concentration corresponding to that used *in vivo*. The study revealed that LLC cell incubation in the presence of native RNase A resulted in a two- to three-fold increase in miRNA content in the cells (Figure 4C). DEPC-inactivated RNase A (no ribonuclease activity) did not affect miRNA expression (Figure 4C). Thus, the increase in miRNA expression in the tumour tissue and reduction in miRNA levels in the bloodstream were the result of RNase A treatment and the ribonuclease activity of the enzyme was required for the observed effects.

### qPCR analysis of miRNA processing genes

**Figure 5:**
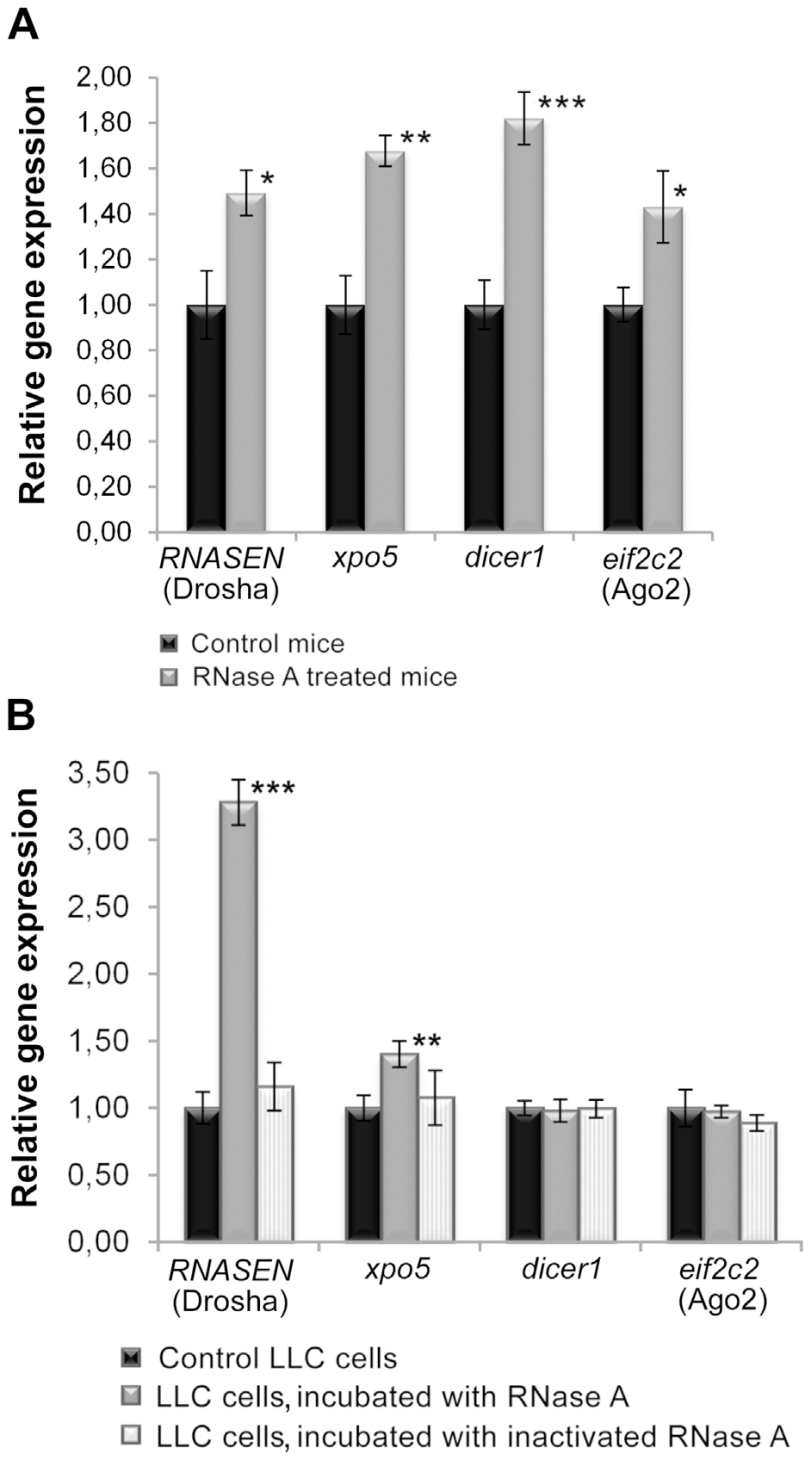
qPCR analysis of the expression of miRNA processing genes. **A**. Expression of miRNA processing genes in LLC tumour tissue from mice that received RNase A therapy. **B**. Expression of miRNA processing genes in LLC cells incubated *in vitro* with intact and DEPC-inactivated RNase A for 48 h. *, **, and *** denote a statistically significant difference relative to the control with *p*<0.05, *p*<0.01 and *p*<0.001, respectively. *hprt1* and *rpl30* were used as reference genes.

The observed RNase A-mediated boost of miRNA synthesis in tumour tissue might be associated with alterations in miRNA transcription or processing machinery. In order to find out at what stage of miRNA synthesis upregulation occurred, we analysed the expression of mRNAs encoding the key molecules known to be important for miRNA processing: RNASEN (Drosha), xpo5, dicer1, and eif2c2 (Ago2). The analysis of qPCR results indicated that all of the studied genes were upregulated in response to *in vivo* RNase A therapy (Figure 5A). The study of gene expression after *in vitro* exposure of tumour cells to native and DEPC-inactivated RNase A showed that the incubation of tumour cells with RNase A for 48 h resulted in a significant increase in the expression of Drosha and xpo5 (Figure 5B), whereas the inactivated RNase A did not affect the miRNA biogenesis machinery.

These data showed that single exposure (for 2 days) of tumour cells to RNase A caused significant activation of the transcription of genes encoding miRNAs and some genes of the miRNA processing machinery. Multiple applications of RNase A *in vivo* (for 10 days) led to the activation of the whole miRNA processing machinery.

Thus, for the first time it was shown that antitumour treatment with RNase A mediates an increase in miRNA expression in tumour cells and a reduction of miRNA level in the bloodstream. Along with tumour miRNA overexpression an upregulation of miRNA processing genes was observed. Ribonuclease activity of RNase A was shown to be crucial for the activation of both microRNA synthesis and expression of the microRNA processing genes.

## Discussion

High-throughput SOLiD™ sequencing technology was applied to analyse genome-wide profiles of miRNAs in the tumour and serum of LLC-bearing mice after multiple i.m. administration of RNase A at the dose provided for antitumour and antimetastatic effects [24]. As we expected based on the high ribonucleolytic activity of RNase A, the drop of miRNA levels in the bloodstream was observed upon RNase A treatment. However, in tumour cells, a global miRNA boost as well as the overexpression of genes encoding the miRNA processing machinery occurred.

Recent studies have revealed that tumour progression is accompanied by enhanced levels of circulating RNAs, and miRNAs originating from tumours are found in the bloodstream. Extracellular miRNAs in the blood are enclosed in small membranous vesicles, such as exosomes, shedding vesicles, and apoptotic bodies, or complexed with RNA-binding proteins, in particular, high-density lipoprotein (HDL) [36], Ago2 [37], [38] and nucleophosmin 1 (NPM1) [39], [40]. The majority (97%) of miRNAs of tumour origin circulate in the blood in complexes with Ago2 [38], which are very stable and protect miRNAs from degradation, similar to small membranous vesicles. We checked the possibility of miRNA cleavage by RNase A in serum under *in vitro* conditions and did not observe miRNA degradation (primary data not shown). Therefore, the question of how RNase A degrades circulating miRNAs remains to be answered.

**Figure 6:**
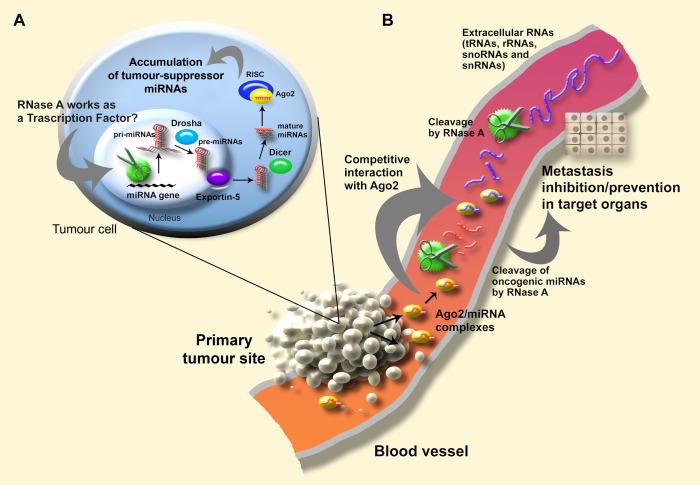
Hypothetical mechanism of the effects of RNase A on the biogenesis of tumour-derived and circulating miRNAs. **A**. In tumour cells, positively charged RNase A binds with negatively charged tumour tissue (due to the presence of anion phosphatidylserine residues on the surface of tumour blood vessels) and penetrate into the cells. RNase A-mediated increase in miRNA levels in tumour cells may be a result of the activity of RNase A or its proteolytic fragment as a transcription activator. Apparently RNase A displays some properties of homologous angiogenin, which was shown to stimulate ribosomal RNA transcription and protein synthesis [46] and thus can possibly act as a transcriptional activator in tumour cells. **B**. The primary tumour site is a source of extracellular nucleoprotein complexes, in particular miRNA/Ago2 complexes [37], [38]. Recent studies have shown a large number of small (∼20–30 nt) RNAs, originated from tRNAs, rRNA, snoRNAs and snRNAs, that are able to associate with Ago2 and perform the regulatory role by RNAi pathway [42]. In the bloodstream RNase A, whose concentration in the bloodstream is maintained by daily intramuscular injections, may generate short fragments of extracellular RNAs (tRNAs, rRNAs, snoRNAs, snRNAs), that may competitively displace miRNAs from nucleoprotein complexes, resulting in the degradation of released miRNAs by RNase A.

Recently, independent groups of authors demonstrated that non-coding RNAs such as tRNAs, snoRNAs, rRNAs, and snRNAs can undergo stress-induced cleavage by RNase III endonuclease Dicer and several members of other RNase families, including angiogenin from the RNase A superfamily, with the generation of stable short RNA products, which are capable of associating with Ago2 [41], [42], [43]. It was shown with the example of tRNA that RNase A (0.4 µg/ml) was able to generate fragments of 20–25 nt in length, similar to angiogenin [41]. Applying these findings to our results, we assume that RNase A may cleave non-coding RNAs in the bloodstream with the generation of a set of short fragments, which can compete with miRNAs for binding with Ago2. This can lead to a displacement of miRNAs from miRNA/Ago2 complexes and its further degradation by RNase A. Repeated administrations of RNase A could result in the maintenance of a constantly elevated concentration of short RNA fragments and, consequently, continuous displacement of miRNAs from newly arrived complexes. The hypothetical mechanism for the effects of RNase A on tumour and serum miRNAs is depicted in Figure 6.

The most intriguing question is how RNase A may stimulate miRNA synthesis in tumour cells. The drop of circulating miRNA levels in the bloodstream was most likely not the reason for the boost in tumour miRNAs, since a similar increase in miRNA expression was observed after exposure of the cells to RNase A *in vitro*. The global boost of miRNAs and elevated expression of genes encoding the miRNA processing machinery in tumour cells may be the result of the activation of specific transcription factors, promoting miRNA expression. Some of the members of the RNase A family can act like transcription factors, for example, angiogenin, which has 33% sequence identity and 65% structure homology to RNase A [44], [45]. It can be speculated that after penetrating into cells, RNase A or its angiogenin-like fragment, formed after proteolysis *in vivo*, may act as a transcription factor (Figure 6). Angiogenin has been shown to bind to the promoter region of ribosomal DNA (rDNA) and stimulate rRNA transcription and protein synthesis [46]. An enhanced expression of 18S rRNA in tumour cells (primary data not shown) that was detected after multiple administrations of RNase A suggests the possible function of RNase A as a transcriptional activator.

Generally, the overproduction of angiogenin is a negative event in tumourigenesis, due to its ability to trigger neovascularisation [47], [48], cell proliferation [49], and migration [50]. One of the important structural differences between RNase A and angiogenin is the absence of a fourth disulphide bond in angiogenin [51]. The missing disulphide bond in angiogenin that forms part of its cell-binding region has a functional consequence – the ability to stimulate neovascularisation [52]–[54]. Thus, the positively charged RNase A lacks the ability to stimulate angiogenesis but is able to bind with negatively charged tumour tissue [55] and act as a transcriptional activator.

Our data show the importance of the ribonucleolytic function of RNase A, since DEPC-inactivated RNase A did not induce miRNA boost and activation of miRNA biogenesis in tumour cells. In support of our data are the results from the studies with variants and inhibitors of angiogenin that demonstrated the importance of its ribonucleolytic activity in its angiogenic activity [56], [57].

Recent studies indicate that abrogation of global miRNA processing enhances cellular transformation and tumourigenesis [58]. A global decrease in mature miRNA generation is detected in different types of cancers [59], [60]. Karube et al. found that Drosha and specifically Dicer expression levels were reduced in patients with non-small-cell-lung cancer (NSCLC) that correlated with poorly differentiated tumour status and shortened postoperative survival [61]. Thus, RNase A-mediated enhancement in mature miRNA expression may be a beneficial event providing a tumour-suppressive function and reducing tumour malignancy.

Among cytotoxic ribonucleases the ability to affect miRNA biogenesis was shown for Onconase. In malignant pleural mesothelioma cell lines onconase significantly upregulated *hsa*-miR-17 and downregulated *hsa*-mir-30c that resulted in NFκ-β inhibition and an increase in chemosensitivity of tumour cells [29]. Another *in vitro* study in mesothelioma cell line Msto-211h showed that onconase downregulated intracellular miRNAs by cleavage of miRNA precursors [30]. RNase A being tightly bound by ribonuclease inhibitor inside the cell did not execute direct RNA cleavage but caused global miRNA boost due to transcription activation.

It is worth noting that in general the entire pattern of upregulated tumour-derived miRNAs is not unambiguous because we observed the activation of both tumour-suppressor (miRNAs of let-7 family, mir-451) and oncogenic miRNAs (mir-29b, mir-21). However among the top 100 upregulated tumour-derived miRNAs there are tumour-suppressors, in particular, miRNAs let-7 family (11 of 12 in the mammalian let-7 family, Figure 3) known to negatively regulate events of tumour progression [62], [63] and be important for cancer patient survival [64]. Thus despite the activation by RNase A of the synthesis of oncogenic miRNAs along with tumour-suppressor miRNAs the total effect of miRNA repertoire rearrangement brings to the reduction of tumour malignancy resulting in inhibition of tumour growth and metastasis.

## Supporting Information

Table S1 Specific RT stem-loop primers. For the design of specific RT-primers, we used a loop sequence taken from [35].
(DOCX)

Table S2 PCR primers. PCR primers for *Drosha*, *xpo5*, *dicer1*, *eif2c2*, *rpl30*, and *hprt1* were designed using NCBI Primer-BLAST program and OligoAnalyzer 3.1. The forward primer for *U6* was taken from [65].
(DOCX)

Table S3 Abundance of microRNA (miRNA) in the cDNA libraries L1 and L2, constructed on the basis of the short RNA fraction from the tumour tissue of mice with LLC treated with saline buffer or RNase A. The table shows miRNAs that are statistically significantly changed in the tumour tissue of mice with LLC after the treatment with RNase A. L1 – cDNA library constructed on the basis of the short RNA fraction from the tumour tissue of mice with LLC treated with saline buffer (RNA_STc_); L2 – cDNA library constructed on the basis of the short RNA fraction from the tumour tissue of mice with LLC treated with RNase A (RNA_STR_). ^*^RPKM (reads per kb per million) = number of reads of specific miRNA/(size of miRNA(kb) x total number of reads in library(mln)). ^#^Fold change = RPKM_L2/RPKM_L1, ^##^Fold change = RPKM_L1/RPKM_L2. miRNAs specific for both the tumour and serum are highlighted in light purple. miRNAs specific for the tumour only are highlighted in light blue.
(DOCX)

Table S4 Abundance of miRNA in the cDNA libraries constructed on the base of short RNA fractions from the blood serum of mice with LLC treated with saline buffer or RNase A. The table shows miRNAs that are significantly changed in the blood serum of mice with LLC after treatment with RNase A. L3 – cDNA library constructed on the basis of the short RNA fraction from the blood serum of mice with LLC treated with saline buffer (RNA_SSc_); L4 – cDNA library constructed on the basis of the short RNA fraction from the blood serum of mice with LLC treated with RNase A (RNA_SSR_). ^*^RPKM (reads per kb per million) = number of reads of specific miRNA/(size of miRNA(kb) x total number of reads in library(mln)). ^#^Fold change = RPKM_L3/RPKM_L4, ^##^Fold change = RPKM_L4/RPKM_L3. miRNAs specific for both the tumour and serum are highlighted in light purple. miRNAs specific for the serum only are highlighted in pink.
(DOCX)

Table S5 Criteria of miRNA selection. The algorithm for miRNA selection consisted of the following steps: miRNAs of the L1 library were sorted by abundance score in descending order and the data of the L2 library were superposed. In a similar way, miRNAs of the L3 library were sorted by abundance score in descending order and the data of the L4 library were superposed. The L2/L1 and L3/L4 fold changes were calculated. The top 100 miRNAs of the L1 library with superposed L2 data were selected and sorted by L2/L1 fold change in descending order, and then the data of L3/L4 were superposed. At this step, serum-specific miRNAs that were not discovered in tumour tissue were rejected. Thus, further miRNAs presented in all libraries were analysed. Total score (TS) was calculated as TS = score in L1+ score in L3 and the data were sorted by total score in descending order. Total fold (TF) was calculated as TF = L2/L1 fold change +L3/L4 fold change and the data were sorted by total fold in descending order. The ratio between TS and TF (TS/TF) was calculated and the data were sorted by TS/TF in ascending order. miRNAs with high TF and TS/TF between 0 to 20 were considered as potential targets for validation. Nine of these miRNAs, which were known to be oncomirs or oncosuppressors according to data in the literature, were selected for validation using qPCR.
(DOCX)
