# Supplementary material for: MicroRNA Drop in the Bloodstream and MicroRNA Boost in the Tumour Caused by Treatment with Ribonuclease A Leads to an Attenuation of Tumour Malignancy
Source: PLoS One. 2013 Dec 30;8(12):e83482. doi: 10.1371/journal.pone.0083482 (PMC3875445; doi:10.1371/journal.pone.0083482)
Supplement: Table S1 — Specific RT stem-loop primers. For the design of specific RT-primers, we used a loop sequence taken from [35]. (DOCX) [file pone.0083482.s001.docx]

**Table S1.**

| **Name** | **RT-primer sequence, 5’ → 3’** |
| --- | --- |
| *mmu*-mir-18a-5p | GTCGTATCCAGTGCAGGGTCCGAGGTATTCGCACTGGATACGACTATCTGCACT |
| *mmu*-mir-17-5p | GTCGTATCCAGTGCAGGGTCCGAGGTATTCGCACTGGATACGACCTACCTGCAC |
| *mmu*-mir-31-5p | GTCGTATCCAGTGCAGGGTCCGAGGTATTCGCACTGGATACGACCAGCTATGCC |
| *mmu*-mir-29-b-1-5p | GTCGTATCCAGTGCAGGGTCCGAGGTATTCGCACTGGATACGACTAAACCACCA |
| *mmu*-mir-145a-5p | GTCGTATCCAGTGCAGGGTCCGAGGTATTCGCACTGGATACGACAGGGATTCCT |
| *mmu*-mir-451a-5p | GTCGTATCCAGTGCAGGGTCCGAGGTATTCGCACTGGATACGACAACTCAGTAA |
| *mmu*-mir-10b-5p | GTCGTATCCAGTGCAGGGTCCGAGGTATTCGCACTGGATACGACCACAAATTCG |
| *mmu*-mir-21-5p | GTCGTATCCAGTGCAGGGTCCGAGGTATTCGCACTGGATACGACTCAACATCAG |
| *mmu*-let-7g-5p | GTCGTATCCAGTGCAGGGTCCGAGGTATTCGCACTGGATACGACAACTGTACAA |
| *U6* | GTCGTATCCAGTGCAGGGTCCGAGGTATTCGCACTGGATACGACAAAAATATGGAACG |
