# Supplementary material for: MicroRNA Drop in the Bloodstream and MicroRNA Boost in the Tumour Caused by Treatment with Ribonuclease A Leads to an Attenuation of Tumour Malignancy
Source: PLoS One. 2013 Dec 30;8(12):e83482. doi: 10.1371/journal.pone.0083482 (PMC3875445; doi:10.1371/journal.pone.0083482)
Supplement: Table S2 — PCR primers. PCR primers for Drosha, xpo5, dicer1, eif2c2, rpl30, and hprt1 were designed using NCBI Primer-BLAST program and OligoAnalyzer 3.1. The forward primer for U6 was taken from [65]. (DOCX) [file pone.0083482.s002.docx]

**Table S2.**

| **Name** | **Forward primer sequence, 5’ → 3’** | **Reverse primer sequence, 5 ’→ 3’** |
| --- | --- | --- |
| *mmu*-mir-18a-5p | GCGTAAGGTGCATCTAGTG | GTGCAGGGTCCGAGGT |
| *mmu*-mir-17-5p | AGACAAAGTGCTTACAGTGC | GTGCAGGGTCCGAGGT |
| *mmu*-mir-31-5p | AGGCAAGATGCTGGCA | GTGCAGGGTCCGAGGT |
| *mmu*-mir-29-b-1-5p | AACGCTGGTTTCATATGGT | GTGCAGGGTCCGAGGT |
| *mmu*-mir-145a-5p | AGGTCCAGTTTTCCCAGGA | GTGCAGGGTCCGAGGT |
| *mmu*-mir-451a-5p | ACGCAAACCGTTACCATTAC | GTGCAGGGTCCGAGGT |
| *mmu*-mir-10b-5p | TACCCTGTAGAACCGAA | GTGCAGGGTCCGAGGT |
| *mmu*-mir-21-5p | AGACTAGCTTATCAGACTGA | GTGCAGGGTCCGAGGT |
| *mmu*-let-7g-5p | AACGCTGAGGTAGTAGTTTGT | GTGCAGGGTCCGAGGT |
| *U6* | CTCGCTTCGGCAGCACA | GTGCAGGGTCCGAGGT |
| *drosha* | ATGAAAGGAGCAGAGAGCGG | CATCTCCCCAGGCAACTCTG |
| *xpo5* | GGCATGAAGTTCTGTCCCGT | TCGGGAGGAGTTGAAGAAAGC |
| *dicer1* | CAAACAGATGGAGGCGGAGT | ATTAGAGATCGGCGCTCGTG |
| *eif2c2* | TCCATCAAGTGGGTGTCGTG | GGGTTGGAACAGCCTTCAGA |
| *rpl30* | CTCTCTTCTGTCCTCTGTGTAT | AAGGATAACCAACTTCGCTTTG |
| *hprt1* | TTGCTGACCTGCTGGATTAC | AGTTGAGAGATCATCTCCAC |
