# Supplementary material for: MicroRNA Drop in the Bloodstream and MicroRNA Boost in the Tumour Caused by Treatment with Ribonuclease A Leads to an Attenuation of Tumour Malignancy
Source: PLoS One. 2013 Dec 30;8(12):e83482. doi: 10.1371/journal.pone.0083482 (PMC3875445; doi:10.1371/journal.pone.0083482)
Supplement: Table S3 — Abundance of microRNA (miRNA) in the cDNA libraries L1 and L2, constructed on the basis of the short RNA fraction from the tumour tissue of mice with LLC treated with saline buffer or RNase A. The table shows miRNAs that are statistically significantly changed in the tumour tissue of mice with LLC after the treatment with RNase A. L1 – cDNA library constructed on the basis of the short RNA fraction from the tumour tissue of mice with LLC treated with saline buffer (RNASTc); L2 – cDNA library constructed on the basis of the short RNA fraction from the tumour tissue of mice with LLC treated with RNase A (RNASTR). *RPKM (reads per kb per million) = number of reads of specific miRNA/(size of miRNA(kb) x total number of reads in library(mln)). #Fold change = RPKM_L2/RPKM_L1, ##Fold change = RPKM_L1/RPKM_L2. miRNAs specific for both the tumour and serum are highlighted in light purple. miRNAs specific for the tumour only are highlighted in light blue. (DOCX) [file pone.0083482.s003.docx]

**Table S3.**

| **Score in L1** | **miRNA** | **value_L1, RPKM^*^** | **value_L2, RPKM^*^** | **Fold change^#^** |
| --- | --- | --- | --- | --- |
| **1** | *mmu-*mir-21a | 663960 | 1.5^.^10^6^ | 2.2 |
| **2** | *mmu-*mir-451a | 197175 | 629581 | 3.2 |
| **3** | *mmu-*mir-125a | 193444 | 496480 | 2.6 |
| **4** | *mmu-*mir-191 | 148778 | 244460 | 1.6 |
| **5** | *mmu-*mir-23a | 144962 | 307627 | 2.2 |
| **6** | *mmu-*mir-130a | 142445 | 294427 | 2.1 |
| **7** | *mmu-*mir-126 | 139759 | 242306 | 1.7 |
| **8** | *mmu-*mir-17 | 112977 | 197623 | 1.7 |
| **9** | *mmu-*mir-140 | 110787 | 175576 | 1.6 |
| **10** | *mmu-*mir-145 | 110102 | 161281 | 1.5 |
| **11** | *mmu*-mir-23b | 89367.7 | 201004 | 2.3 |
| **12** | *mmu*-mir-99b | 71637 | 131304 | 1.8 |
| **13** | *mmu*-mir-107 | 69363.5 | 106212 | 1.5 |
| **14** | *mmu*-mir-15b | 63458.2 | 104735 | 1.7 |
| **15** | *mmu*-mir-31 | 59155.6 | 189672 | 3.2 |
| **16** | *mmu*-mir-93 | 46018 | 86762.8 | 1.9 |
| **17** | *mmu*-mir-27a | 45753.6 | 97540.6 | 2.1 |
| **18** | *mmu*-mir-25 | 34458.6 | 71508.4 | 2.1 |
| **19** | *mmu*-mir-143 | 29087 | 57932 | 2.0 |
| **20** | *mmu*-mir-130b | 28336.5 | 60497.8 | 2.1 |
| **21** | *mmu*-mir-18a | 27846 | 122258 | 4.4 |
| **22** | *mmu*-mir-99a | 23767 | 13807.7 | -1.7^##^ |
| **23** | *mmu-*mir-301 | 21209.2 | 61342.4 | 2.9 |
| **24** | *mmu*-mir-138-2 | 21075.8 | 45326.3 | 2.2 |
| **25** | *mmu*-mir-199b | 20850.2 | 67454 | 3.2 |
| **26** | *mmu*-let-7b | 19200.2 | 37405.3 | 2.0 |
| **27** | *mmu*-mir-29b-1 | 17499 | 68431 | 3.9 |
| **28** | *mmu*-mir-125b-1 | 17042.3 | 43897.3 | 2.6 |
| **29** | *mmu*-mir-10b | 16617.6 | 51173.4 | 3.1 |
| **30** | *mmu*-mir-222 | 16506.6 | 33401.2 | 2.0 |
| **31** | *mmu*-mir-221 | 15996 | 24781 | 1.6 |
| **32** | *mmu*-mir-19b-1 | 15450.7 | 38213.2 | 2.5 |
| **33** | *mmu*-mir-20a | 14924.3 | 53026 | 3.6 |
| **34** | *mmu*-mir-19b-2 | 14630.7 | 50329.6 | 3.5 |
| **35** | *mmu-*mir-19a | 13171.3 | 64012 | 4.9 |
| **36** | *mmu-*mir-421 | 12988.4 | 40445 | 3.1 |
| **37** | *mmu*-let-7i | 12197.8 | 32186.3 | 2.6 |
| **38** | *mmu*-mir-138-1 | 10474 | 18905 | 1.8 |
| **39** | *mmu*-mir-872 | 1019.6 | 16588.2 | 1.6 |
| **40** | *mmu*-mir-532 | 1015.8 | 15340.4 | 1.5 |
| **41** | *mmu*-mir-132 | 9561 | 24092.3 | 2.5 |
| **42** | *mmu*-mir-193 | 9463.8 | 34518.6 | 3.7 |
| **43** | *mmu*-mir-425 | 8486.8 | 13615.4 | 1.6 |
| **44** | *mmu*-mir-30e | 8041.7 | 18100 | 2.3 |
| **45** | *mmu*-mir-15a | 7801.5 | 17192.8 | 2.2 |
| **46** | *mmu*-let-7g | 7419.8 | 26008.2 | 3.5 |
| **47** | *mmu*-mir-155 | 7309 | 24016.8 | 3.3 |
| **48** | *mmu*-let-7d | 6891.6 | 15629 | 2.3 |
| **49** | *mmu*-mir-27b | 6857.8 | 15717.7 | 2.3 |
| **50** | *mmu*-mir-125b-2 | 6692 | 19013.5 | 2.8 |
| **51** | *mmu*-mir-92-2 | 6437.5 | 13638.7 | 2.1 |
| **52** | *mmu*-mir-30d | 5909 | 14944 | 2.5 |
| **53** | *mmu*-mir-1839 | 5802.8 | 16659.7 | 2.9 |
| **54** | *mmu*-mir-3068 | 5793 | 10973.8 | 1.9 |
| **55** | *mmu-*mir-150 | 5699 | 1799.3 | -3.2^##^ |
| **56** | *mmu*-mir-484 | 5377.3 | 11458.6 | 2.1 |
| **57** | *mmu*-mir-139 | 4883.6 | 3010.2 | -1.6^##^ |
| **58** | *mmu*-mir-503 | 4788.2 | 7623 | 1.6 |
| **59** | *mmu*-let-7f-2 | 4485 | 23047.8 | 5.1 |
| **60** | *mmu*-mir-3074-2 | 4287.7 | 7042.4 | 1.6 |
| **61** | *mmu*-let-7e | 4118.3 | 16809 | 4.1 |
| **62** | *mmu*-mir-185 | 4114.5 | 6454 | 1.6 |
| **63** | *mmu*-mir-16-1 | 4070.2 | 8785.5 | 2.1 |
| **64** | *mmu*-mir-34c | 3494.3 | 9473 | 2.7 |
| **65** | *mmu*-mir-322 | 3280 | 9857.4 | 3.0 |
| **66** | *mmu*-mir-144 | 3211.4 | 12921.2 | 4.0 |
| **67** | *mmu-*mir-10a | 3137.7 | 5849.3 | 1.9 |
| **68** | *mmu*-mir-186 | 3134.5 | 8918 | 2.9 |
| **69** | *mmu*-mir-374c | 3052 | 22359 | 7.3 |
| **70** | *mmu*-mir-320 | 2827.6 | 9247.6 | 3.3 |
| **71** | *mmu-*mir-1949 | 2777.7 | 11060 | 4.0 |
| **72** | *mmu*-let-7f-1 | 2742.3 | 7558 | 2.8 |
| **73** | *mmu*-mir-345 | 2583.4 | 4807 | 1.9 |
| **74** | *mmu*-mir-192 | 2473.6 | 5501.8 | 2.2 |
| **75** | *mmu*-mir-1-1 | 2369 | 6504 | 2.7 |
| **76** | *mmu*-mir-26b | 2076 | 4461.6 | 2.2 |
| **77** | *mmu*-mir-362 | 1907.5 | 6949.5 | 3.6 |
| **78** | *mmu*-mir-674 | 1614 | 3536.2 | 2.2 |
| **79** | *mmu-*let-7a-1 | 1573.7 | 3512.2 | 2.2 |
| **80** | *mmu*-let-7c-1 | 1519.5 | 4805.6 | 3.2 |
| **81** | *mmu*-mir-101b | 1518 | 5813.6 | 3.8 |
| **82** | *mmu*-mir-34b | 1453 | 3490 | 2.4 |
| **83** | *mmu*-mir-500 | 1430.4 | 3284 | 2.3 |
| **84** | *mmu*-mir-101a | 1417.5 | 5993 | 4.2 |
| **85** | *mmu*-mir-7-1 | 1242.4 | 2262.4 | 1.8 |
| **86** | *mmu*-mir-127 | 1161.4 | 148 | -7.8^##^ |
| **87** | *mmu*-mir-212 | 1111.7 | 2071 | 1.9 |
| **88** | *mmu*-mir-542 | 1022 | 4083 | 4.0 |
| **89** | *mmu*-mir-365-1 | 1017.4 | 3633.5 | 3.6 |
| **90** | *mmu-*mir-449a | 836 | 3482.2 | 4.2 |
| **91** | *mmu*-mir-148a | 817.8 | 370.5 | -2.2^##^ |
| **92** | *mmu*-mir-188 | 817.6 | 2164.6 | 2.7 |
| **93** | *mmu*-mir-350 | 798 | 1772.4 | 2.2 |
| **94** | *mmu*-mir-196a-2 | 699.2 | 2601.3 | 3.7 |
| **95** | *mmu*-mir-511 | 681.5 | 1335.2 | 2.0 |
| **96** | *mmu*-mir-205 | 663 | 169 | -3.9^##^ |
| **97** | *mmu*-mir-331 | 651.3 | 2182 | 3.4 |
| **98** | *mmu*-mir-328 | 538.6 | 1124.2 | 2.1 |
| **99** | *mmu*-let-7c-2 | 464.3 | 1008.7 | 2.2 |
| **100** | *mmu*-let-7a-2 | 448.7 | 1229.4 | 2.7 |
| **101** | *mmu*-mir-615 | 432.4 | 802 | 1.9 |
| **102** | *mmu*-mir-582 | 418.8 | 1141.8 | 2.7 |
| **103** | *mmu*-mir-181c | 417 | 1021 | 2.5 |
| **104** | *mmu*-mir-18b | 406.7 | 2436.4 | 6.0 |
| **105** | *mmu-*mir-449c | 390.6 | 1057 | 2.7 |
| **106** | *mmu*-mir-330 | 385.5 | 911 | 2.4 |
| **107** | *mmu*-mir-196a-1 | 373.6 | 844.2 | 2.3 |
| **108** | *mmu*-mir-505 | 339.5 | 875 | 2.6 |
| **109** | *mmu*-mir-411 | 302 | 74 | -4.1^##^ |
| **110** | *mmu*-mir-1843 | 260 | 1574 | 6.1 |
| **111** | *mmu*-mir-7-2 | 140 | 481.8 | 3.4 |
| **112** | *mmu*-mir-1249 | 135.3 | 455.5 | 3.4 |
| **113** | *mmu*-mir-134 | 96 | 588.7 | 6.1 |
| **114** | *mmu*-mir-181d | 92 | 478.3 | 5.2 |
| **115** | *mmu*-mir-1-2 | 68.7 | 190.3 | 2.8 |
| **116** | *mmu*-mir-501 | 57.2 | 291.6 | 5.1 |
| **117** | *mmu*-mir-1199 | 28.5 | 141.5 | 5.0 |
| **118** | *mmu*-mir-718 | 27.4 | 312.3 | 11.4 |
| **119** | *mmu*-mir-1965 | 8.8 | 58.4 | 6.8 |
| **120** | *mmu*-mir-1969 | 0 | 71.2 |  |
| **121** | *mmu*-mir-3061 | 0 | 79 |  |
| **122** | *mmu*-mir-743 | 0 | 956.8 |  |
| **123** | *mmu*-mir-743b | 0 | 226.6 |  |
