# Supplementary material for: MicroRNA Drop in the Bloodstream and MicroRNA Boost in the Tumour Caused by Treatment with Ribonuclease A Leads to an Attenuation of Tumour Malignancy
Source: PLoS One. 2013 Dec 30;8(12):e83482. doi: 10.1371/journal.pone.0083482 (PMC3875445; doi:10.1371/journal.pone.0083482)
Supplement: Table S4 — Abundance of miRNA in the cDNA libraries constructed on the base of short RNA fractions from the blood serum of mice with LLC treated with saline buffer or RNase A. The table shows miRNAs that are significantly changed in the blood serum of mice with LLC after treatment with RNase A. L3 – cDNA library constructed on the basis of the short RNA fraction from the blood serum of mice with LLC treated with saline buffer (RNASSc); L4 – cDNA library constructed on the basis of the short RNA fraction from the blood serum of mice with LLC treated with RNase A (RNASSR). *RPKM (reads per kb per million) = number of reads of specific miRNA/(size of miRNA(kb) x total number of reads in library(mln)). #Fold change = RPKM_L3/RPKM_L4, ##Fold change = RPKM_L4/RPKM_L3. miRNAs specific for both the tumour and serum are highlighted in light purple. miRNAs specific for the serum only are highlighted in pink. (DOCX) [file pone.0083482.s004.docx]

**Table S4.**

| **Score in L3** | **miRNA** | **value_L3, RPKM^*^** | **value_L4, RPKM^*^** | **Fold change^#^** |
| --- | --- | --- | --- | --- |
| **1** | *mmu*-mir-29a | 1309760 | 307410 | 4.3 |
| **2** | *mmu*-mir-451a | 1043800 | 287144 | 3.6 |
| **3** | *mmu*-mir-140 | 869954 | 486671 | 1.8 |
| **4** | *mmu*-mir-23a | 604959 | 222958 | 2.7 |
| **5** | *mmu*-mir-145 | 384463 | 168195 | 2.3 |
| **6** | *mmu*-mir-205 | 277686 | 113413 | 2.5 |
| **7** | *mmu*-mir-130a | 215001 | 62320.4 | 3.5 |
| **8** | *mmu*-mir-15b | 198555 | 92987.2 | 2.1 |
| **9** | *mmu*-mir-99a | 191884 | 77512.6 | 2.5 |
| **10** | *mmu*-mir-23b | 184451 | 67119 | 2.8 |
| **11** | *mmu*-mir-142 | 162985 | 40459 | 4.0 |
| **12** | *mmu*-mir-125a | 157965 | 61373.7 | 2.6 |
| **13** | *mmu*-mir-126 | 118654 | 36665.4 | 3.24 |
| **14** | *mmu*-mir-320 | 96308.6 | 43308.5 | 2.2 |
| **15** | *mmu*-mir-484 | 75419 | 45546 | 1.7 |
| **16** | *mmu*-mir-27a | 71993 | 24095.8 | 3.0 |
| **17** | *mmu*-mir-425 | 71773 | 37168.4 | 1.9 |
| **18** | *mmu*-mir-10b | 66826.3 | 17919 | 3.7 |
| **19** | *mmu*-mir-328 | 61512.4 | 38717.2 | 1.6 |
| **20** | *mmu*-mir-150 | 55039.4 | 26210.6 | 2.1 |
| **21** | *mmu*-mir-149 | 51604.6 | 36499.9 | 1.4 |
| **22** | *mmu*-mir-92-2 | 50772.8 | 35829 | 1.4 |
| **23** | *mmu*-mir-122a | 49724.5 | 4639.8 | 10.7 |
| **24** | *mmu*-mir-146 | 48906.6 | 13791.3 | 3.6 |
| **25** | *mmu*-mir-221 | 45156.6 | 15086 | 3.0 |
| **26** | *mmu*-mir-193b | 38864.2 | 8607.4 | 4.5 |
| **27** | *mmu-*mir-30b | 35615 | 16592 | 2.2 |
| **28** | *mmu*-mir-187 | 33816 | 23889 | 1.4 |
| **29** | *mmu*-mir-192 | 32576 | 6177.4 | 5.3 |
| **30** | *mmu*-mir-30d | 32070 | 12171.4 | 2.6 |
| **31** | *mmu*-mir-17 | 29981.7 | 11510.7 | 2.6 |
| **32** | *mmu*-mir-139 | 26245.2 | 17700.3 | 1.5 |
| **33** | *mmu*-let-7d | 25963.8 | 11034 | 2.4 |
| **34** | *mmu*-mir-193 | 23551 | 5842.7 | 4.0 |
| **35** | *mmu*-mir-1839 | 23244.5 | 4769.5 | 4.9 |
| **36** | *mmu*-mir-100 | 23156.7 | 6785.6 | 3.4 |
| **37** | *mmu*-mir-1-1 | 22102.2 | 3409.5 | 6.5 |
| **38** | *mmu*-mir-203 | 21734.6 | 5454.4 | 4.0 |
| **39** | *mmu*-mir-21a | 21474.8 | 3075 | 7.0 |
| **40** | *mmu*-mir-133b | 19414 | 10169.6 | 1.9 |
| **41** | *mmu*-mir-130b | 18726 | 6026 | 3.1 |
| **42** | *mmu*-mir-132 | 17366.5 | 8764 | 2.0 |
| **43** | *mmu*-mir-222 | 16909.5 | 7355.8 | 2.3 |
| **44** | *mmu*-mir-125b-1 | 16862.7 | 6609.8 | 2.6 |
| **45** | *mmu*-mir-107 | 15092.3 | 7257 | 2.1 |
| **46** | *mmu*-mir-18a | 14349 | 4672 | 3.1 |
| **47** | *mmu*-mir-27b | 14197 | 3626.8 | 3.9 |
| **48** | *mmu*-mir-10a | 13777.2 | 3779.8 | 3.6 |
| **49** | *mmu*-mir-322 | 13569.8 | 4633.8 | 2.9 |
| **50** | *mmu*-mir-532 | 13510 | 5970.2 | 2.3 |
| **51** | *mmu*-mir-339 | 13407 | 8014.8 | 1.7 |
| **52** | *mmu*-mir-409 | 12585 | 5488 | 2.3 |
| **53** | *mmu*-mir-138-2 | 11341.2 | 5623.3 | 2.0 |
| **54** | *mmu*-mir-872 | 10867 | 3978.6 | 2.7 |
| **55** | *mmu*-mir-511 | 10284.3 | 4197.8 | 2.5 |
| **56** | *mmu*-mir-744 | 10105 | 6509.8 | 1.6 |
| **57** | *mmu-*mir-674 | 9783.3 | 6661.8 | 1.5 |
| **58** | *mmu*-mir-30a | 9385.8 | 3667.4 | 2.6 |
| **59** | *mmu*-mir-15a | 9160.5 | 1863.2 | 4.9 |
| **60** | *mmu*-mir-543 | 8982.8 | 2814.5 | 3.2 |
| **61** | *mmu*-let-7b | 8553.3 | 3434.2 | 2.5 |
| **62** | *mmu-*mir-185 | 7588 | 2563.3 | 3.0 |
| **63** | *mmu*-mir-200b | 6999.9 | 1763 | 4.0 |
| **64** | *mmu*-mir-128-2 | 6815.6 | 2923.6 | 2.3 |
| **65** | *mmu*-mir-500 | 6596 | 3289.5 | 2.0 |
| **66** | *mmu*-mir-676 | 5784.2 | 3206.5 | 1.8 |
| **67** | *mmu*-mir-382 | 5747.7 | 2138 | 2.7 |
| **68** | *mmu*-mir-19b-2 | 5682.8 | 1468.4 | 3.9 |
| **69** | *mmu-*mir-144 | 5492 | 1374.8 | 4.0 |
| **70** | *mmu*-mir-365-1 | 5314.8 | 2170 | 2.5 |
| **71** | *mmu*-mir-134 | 5084 | 2553.6 | 2.0 |
| **72** | *mmu*-mir-494 | 5009.5 | 790.2 | 6.3 |
| **73** | *mmu*-mir-337 | 4979 | 1097 | 4.5 |
| **74** | *mmu*-mir-34b | 4955.3 | 1089.5 | 4.6 |
| **75** | *mmu*-mir-664 | 4932.2 | 1448.8 | 3.4 |
| **76** | *mmu*-mir-224 | 4546.3 | 1178 | 3.9 |
| **77** | *mmu*-mir-19b-1 | 4422.2 | 1212 | 3.7 |
| **78** | *mmu*-mir-324 | 4314.6 | 2591.4 | 1.7 |
| **79** | *mmu*-mir-212 | 4311.4 | 2836.6 | 1.5 |
| **80** | *mmu*-mir-31 | 4302.7 | 1062.7 | 4.0 |
| **81** | *mmu*-mir-421 | 4114.5 | 1221.8 | 3.4 |
| **82** | *mmu-*mir-215 | 4031 | 766.5 | 5.3 |
| **83** | *mmu*-mir-20a | 3862.2 | 565.2 | 6.8 |
| **84** | *mmu*-mir-34a | 3847.5 | 2002.4 | 1.9 |
| **85** | *mmu*-mir-503 | 3754.3 | 2091.8 | 1.8 |
| **86** | *mmu*-mir-485 | 3719.1 | 1018.5 | 3.7 |
| **87** | *mmu*-mir-143 | 3449.2 | 998.3 | 3.5 |
| **88** | *mmu*-mir-667 | 3433.3 | 2197 | 1.6 |
| **89** | *mmu-*mir-326 | 3259.2 | 1967.8 | 1.7 |
| **90** | *mmu*-mir-30e | 3112 | 1010.4 | 3.1 |
| **91** | *mmu-*mir-204 | 2921 | 1061.4 | 2.8 |
| **92** | *mmu*-mir-19a | 2899.8 | 478 | 6.1 |
| **93** | *mmu*-mir-497 | 2886.8 | 1737 | 1.7 |
| **94** | *mmu*-mir-148a | 2754 | 1275.4 | 2.2 |
| **95** | *mmu*-mir-34c | 2650 | 1234 | 2.2 |
| **96** | *mmu*-let-7f-1 | 2619 | 562.8 | 4.7 |
| **97** | *mmu-*mir-350 | 2581 | 582.3 | 4.4 |
| **98** | *mmu*-mir-125b-2 | 2463.8 | 760.6 | 3.2 |
| **99** | *mmu*-mir-296 | 2273 | 847.3 | 2.7 |
| **100** | *mmu*-mir-206 | 2217.2 | 695.6 | 3.2 |
| **101** | *mmu*-mir-299 | 2012 | 599 | 3.4 |
| **102** | *mmu*-let-7g | 1915.5 | 502.4 | 3.8 |
| **103** | *mmu*-mir-541 | 1907.7 | 303 | 6.3 |
| **104** | *mmu*-let-7i | 1881.3 | 775 | 2.4 |
| **105** | *mmu*-mir-200c | 1758 | 774.4 | 2.3 |
| **106** | *mmu*-mir-1943 | 1716.5 | 74.5 | 23.0 |
| **107** | *mmu*-mir-128-1 | 1678.3 | 739.3 | 2.3 |
| **108** | *mmu*-mir-1930 | 1659.3 | 647.4 | 2.6 |
| **109** | *mmu*-mir-877 | 1640.7 | 911.7 | 1.8 |
| **110** | *mmu*-mir-20b | 1597 | 536.2 | 3.0 |
| **111** | *mmu*-mir-186 | 1564.3 | 135.8 | 11.5 |
| **112** | *mmu*-mir-434 | 1560.5 | 403 | 3.9 |
| **113** | *mmu*-mir-103-2 | 1453.2 | 395 | 3.7 |
| **114** | *mmu*-mir-29b-1 | 1408 | 326 | 4.3 |
| **115** | *mmu*-mir-7-1 | 1398.2 | 582.7 | 2.4 |
| **116** | *mmu-*mir-16-1 | 1240.8 | 465 | 2.7 |
| **117** | *mmu*-mir-133a-1 | 1213.4 | 312.2 | 4.0 |
| **118** | *mmu*-mir-200a | 1144.6 | 315.5 | 3.6 |
| **119** | *mmu-*mir-301 | 1032 | 307.2 | 3.4 |
| **120** | *mmu*-mir-874 | 1005 | 2640 | -2.6^##^ |
| **121** | *mmu*-mir-182 | 983.4 | 205 | 4.8 |
| **122** | *mmu*-mir-219-1 | 930.4 | 339.5 | 2.7 |
| **123** | *mmu*-mir-199b | 911.6 | 261 | 3.5 |
| **124** | *mmu*-mir-411 | 909.3 | 102.4 | 8.9 |
| **125** | *mmu*-let-7a-1 | 883 | 175.2 | 5.0 |
| **126** | *mmu*-mir-329 | 839.3 | 355.8 | 2.4 |
| **127** | *mmu-*mir-1249 | 591.5 | 181.6 | 3.3 |
| **128** | *mmu*-mir-363 | 590 | 136.6 | 4.3 |
| **129** | *mmu*-mir-129-2 | 527 | 176.7 | 3.0 |
| **130** | *mmu*-let-7c-1 | 457.2 | 87.6 | 5.2 |
| **131** | *mmu*-mir-101a | 449 | 65.7 | 6.8 |
| **132** | *mmu*-mir-429 | 401.7 | 82 | 4.9 |
| **133** | *mmu*-mir-715 | 347.7 | 835.6 | -2.4^##^ |
| **134** | *mmu*-mir-195 | 315.3 | 65.7 | 4.8 |
| **135** | *mmu*-mir-743b | 241 | 0 |  |
| **136** | *mmu*-mir-3101 | 234.6 | 0 |  |
| **137** | *mmu*-mir-218-1 | 216.2 | 26 |  |
| **138** | *mmu-*mir-802 | 113.8 | 0 |  |
| **139** | *mmu*-mir-196a-1 | 108.6 | 0 |  |
