# Supplementary material for: MicroRNA Drop in the Bloodstream and MicroRNA Boost in the Tumour Caused by Treatment with Ribonuclease A Leads to an Attenuation of Tumour Malignancy
Source: PLoS One. 2013 Dec 30;8(12):e83482. doi: 10.1371/journal.pone.0083482 (PMC3875445; doi:10.1371/journal.pone.0083482)
Supplement: Table S5 — Criteria of miRNA selection. The algorithm for miRNA selection consisted of the following steps: miRNAs of the L1 library were sorted by abundance score in descending order and the data of the L2 library were superposed. In a similar way, miRNAs of the L3 library were sorted by abundance score in descending order and the data of the L4 library were superposed. The L2/L1 and L3/L4 fold changes were calculated. The top 100 miRNAs of the L1 library with superposed L2 data were selected and sorted by L2/L1 fold change in descending order, and then the data of L3/L4 were superposed. At this step, serum-specific miRNAs that were not discovered in tumour tissue were rejected. Thus, further miRNAs presented in all libraries were analysed. Total score (TS) was calculated as TS = score in L1+ score in L3 and the data were sorted by total score in descending order. Total fold (TF) was calculated as TF = L2/L1 fold change +L3/L4 fold change and the data were sorted by total fold in descending order. The ratio between TS and TF (TS/TF) was calculated and the data were sorted by TS/TF in ascending order. miRNAs with high TF and TS/TF between 0 to 20 were considered as potential targets for validation. Nine of these miRNAs, which were known to be oncomirs or oncosuppressors according to data in the literature, were selected for validation using qPCR. (DOCX) [file pone.0083482.s005.docx]

**Table S5.**

| **miRNA** | **Score**  **in L1** | **Score**  **in L3** | **Total score (TS)** | **fold change**  **L2/L1** | **fold change**  **L3/L4** | **Total fold (TF)** | **TS/TF** |
| --- | --- | --- | --- | --- | --- | --- | --- |
| ***mmu-*mir-451** | 2 | 2 | 4 | 3.2 | 3.6 | 6.8 | 0.6 |
| *mmu-*mir-23a | 5 | 4 | 9 | 2.1 | 2.7 | 4.8 | 1.9 |
| *mmu-*mir-130a | 6 | 7 | 13 | 2.1 | 3.5 | 5.5 | 2.4 |
| *mmu-*mir-125a | 3 | 12 | 15 | 2.6 | 2.6 | 5.1 | 2.9 |
| *mmu-*mir-140 | 9 | 3 | 12 | 1.6 | 1.8 | 3.4 | 3.6 |
| ***mmu-*mir-145** | 10 | 5 | 15 | 1.5 | 2.3 | 3.8 | 4.0 |
| *mmu-*mir-126 | 7 | 13 | 20 | 1.7 | 3.2 | 5.0 | 4.02 |
| *mmu-*mir-23b | 11 | 10 | 21 | 2.3 | 2.8 | 5.0 | 4.2 |
| ***mmu-*mir-21** | 1 | 39 | 40 | 2.2 | 7.0 | 9.2 | 4.3 |
| *mmu-*mir-15b | 14 | 8 | 22 | 1.7 | 2.14 | 3.8 | 5.8 |
| *mmu-*mir-27a | 17 | 16 | 33 | 2.1 | 2.99 | 5.1 | 6.5 |
| ***mmu-*mir-10b** | 29 | 18 | 47 | 3.1 | 3.7 | 6.8 | 6.9 |
| ***mmu-*mir-17** | 8 | 31 | 39 | 1.8 | 2.6 | 4.4 | 8.96 |
| ***mmu-*mir-18** | 21 | 46 | 67 | 4.4 | 3.1 | 7.5 | 8.98 |
| *mmu-*mir-99a | 22 | 9 | 31 | 0.6 | 2.5 | 3.0 | 10.1 |
| *mmu-*mir-20a | 33 | 83 | 116 | 3.6 | 6.8 | 10.4 | 11.2 |
| *mmu-*mir-1839 | 53 | 35 | 88 | 2.9 | 4.9 | 7.7 | 11.4 |
| *mmu-*mir-19a | 35 | 92 | 127 | 4.9 | 6.1 | 10.9 | 11.62 |
| *mmu-*mir-130b | 20 | 41 | 61 | 2.1 | 3.1 | 5.2 | 11.64 |
| *mmu-*mir-1-1 | 75 | 37 | 112 | 2.8 | 6.5 | 9.2 | 12.1 |
| *mmu-*mir-186 | 68 | 111 | 179 | 2.9 | 11.5 | 14.4 | 12.5 |
| ***mmu-*mir-31** | 15 | 80 | 95 | 3.2 | 4.05 | 7.3 | 13.1 |
| *mmu-*mir-192 | 74 | 29 | 103 | 2.2 | 5.3 | 7.5 | 13.7 |
| *mmu-*mir-19b-2 | 34 | 68 | 102 | 3.4 | 3.9 | 7.3 | 13.95 |
| *mmu-*mir-125b-1 | 28 | 44 | 72 | 2.6 | 2.6 | 5.1 | 14.04 |
| *mmu-*mir-15a | 45 | 59 | 104 | 2.2 | 4.9 | 7.1 | 14.6 |
| *mmu-*mir-320 | 70 | 14 | 84 | 3.3 | 2.2 | 5.5 | 15.3 |
| *mmu-*mir-27b | 49 | 47 | 96 | 2.3 | 3.9 | 6.2 | 15.5 |
| *mmu-*mir-30d | 52 | 30 | 82 | 2.5 | 2.6 | 5.2 | 15.9 |
| *mmu-*mir-107 | 13 | 45 | 58 | 1.5 | 2.1 | 3.6 | 16.0 |
| *mmu-*mir-144 | 66 | 69 | 135 | 4.0 | 4.0 | 8.0 | 16.8 |
| *mmu-*mir-222 | 30 | 43 | 73 | 2.0 | 2.3 | 4.3 | 16.9 |
| *mmu-*mir-425 | 43 | 17 | 60 | 1.6 | 1.9 | 3.5 | 17.0 |
| ***mmu-*mir-29b-1** | 27 | 114 | 141 | 3.9 | 4.3 | 8.2 | 17.1 |
| *mmu-*let-7d | 48 | 33 | 81 | 2.3 | 2.4 | 4.6 | 17.5 |
| *mmu-*mir-19b-1 | 32 | 77 | 109 | 2.5 | 3.7 | 6.1 | 17.8 |
| *mmu-*mir-421 | 36 | 81 | 117 | 3.1 | 3.4 | 6.5 | 18.0 |
| *mmu-*mir-132 | 41 | 42 | 83 | 2.5 | 2.0 | 4.5 | 18.4 |
| *mmu-*mir-138-2 | 24 | 53 | 77 | 2.2 | 2.0 | 4.2 | 18.48 |
| *mmu-*mir-484 | 56 | 15 | 71 | 2.1 | 1.7 | 3.8 | 18.8 |
| *mmu-*mir-322 | 65 | 49 | 114 | 3.0 | 2.9 | 6.0 | 19.2 |
| *mmu-*mir-101a | 84 | 131 | 215 | 4.2 | 6.8 | 11.1 | 19.4 |
| *mmu-*mir-143 | 19 | 87 | 106 | 2.0 | 3.5 | 5.5 | 19.5 |
| *mmu-*let-7b | 26 | 61 | 87 | 2.0 | 2.5 | 4.4 | 19.6 |
| ***mmu-*let-7g** | 46 | 100 | 146 | 3.5 | 3.8 | 7.3 | 20.0 |
| *mmu-*mir-92-2 | 51 | 22 | 73 | 2.1 | 1.4 | 3.5 | 20.7 |
| *mmu-*mir-10a | 67 | 48 | 115 | 1.9 | 3.6 | 5.5 | 20.9 |
| *mmu-*mir-872 | 39 | 54 | 93 | 1.6 | 2.7 | 4.4 | 21.3 |
| *mmu-*mir-199b | 25 | 123 | 148 | 3.2 | 3.5 | 6.7 | 22.0 |
| *mmu-*mir-34b | 82 | 74 | 156 | 2.4 | 4.6 | 7.0 | 22.5 |
| *mmu-*let-7f-1 | 72 | 95 | 167 | 2.8 | 4.7 | 7.4 | 22.54 |
| *mmu-*mir-301 | 23 | 119 | 142 | 2.9 | 3.4 | 6.3 | 22.7 |
| *mmu-*mir-532 | 40 | 50 | 90 | 1.5 | 2.3 | 3.8 | 23.9 |
| *mmu-*mir-125b-2 | 50 | 98 | 148 | 2.8 | 3.2 | 6.1 | 24.3 |
| *mmu-*let-7c-1 | 80 | 130 | 210 | 3.2 | 5.2 | 8.4 | 25.1 |
| *mmu-*mir-30e | 44 | 90 | 134 | 2.3 | 3.1 | 5.3 | 25.14 |
| *mmu-*mir-365-1 | 89 | 70 | 159 | 3.6 | 2.5 | 6.0 | 26.4 |
| *mmu-*mir-185 | 62 | 62 | 124 | 1.6 | 3.0 | 4.5 | 27.4 |
| *mmu-*let-7i | 37 | 104 | 141 | 2.6 | 2.4 | 5.1 | 27.8 |
| *mmu-*let-7a-1 | 79 | 125 | 204 | 2.2 | 5.0 | 7.3 | 28.1 |
| *mmu-*mir-350 | 93 | 97 | 190 | 2.2 | 4.4 | 6.7 | 28.6 |
| *mmu-*mir-150 | 55 | 20 | 75 | 0.3 | 2.1 | 2.4 | 31.0 |
| *mmu-*mir-328 | 98 | 19 | 117 | 2.1 | 1.6 | 3.7 | 31.8 |
| *mmu-*mir-34c | 64 | 95 | 159 | 2.7 | 2.2 | 4.9 | 32.7 |
| *mmu-*mir-511 | 95 | 55 | 150 | 2.0 | 2.5 | 4.4 | 34.0 |
| *mmu-*mir-500 | 83 | 65 | 148 | 2.3 | 2.0 | 4.3 | 34.4 |
| *mmu-*mir-674 | 78 | 57 | 135 | 2.2 | 1.5 | 3.7 | 36.9 |
| *mmu-*mir-16-1 | 63 | 116 | 179 | 2.2 | 2.7 | 4.8 | 37.1 |
| *mmu-*mir-205 | 96 | 6 | 102 | 0.3 | 2.5 | 2.7 | 37.7 |
| *mmu-*mir-503 | 58 | 85 | 143 | 1.6 | 1.8 | 3.4 | 42.2 |
| *mmu-*mir-139 | 57 | 32 | 89 | 0.6 | 1.5 | 2.1 | 42.4 |
| *mmu-*mir-7-1 | 85 | 115 | 200 | 1.8 | 2.4 | 4.2 | 47.4 |
| *mmu-*mir-212 | 87 | 79 | 166 | 1.9 | 1.5 | 3.4 | 49.1 |
| *mmu-*mir-221 | 31 | 225 | 256 | 1.6 | 3.0 | 4.5 | 56.4 |
| *mmu-*mir-148a | 91 | 94 | 185 | 0.5 | 2.2 | 2.6 | 70.8 |
